# Ovary Structure and Oogenesis of *Trypophloeus klimeschi* (Coleoptera: Curculionidae: Scolytinae)

**DOI:** 10.3390/insects12121099

**Published:** 2021-12-08

**Authors:** Jing Gao, Jiaxing Wang, Hui Chen

**Affiliations:** 1 State Key Laboratory for Conservation and Utilization of Subtropical Agro-Bioresources, College of Forestry and Landscape Architecture, South China Agricultural University, Guangzhou 510642, China; sxllgaojing@nwafu.edu.cn (J.G.); wjx2017@nwafu.edu.cn (J.W.); 2 College of Forestry, Northwest A&F University, Yangling, Xianyang 712100, China

**Keywords:** female reproductive system, telotrophic meroistic ovary, oogenesis, *Trypophloeus klimeschi*, microscopy, tropharium

## Abstract

**Simple Summary:**

Coleoptera is the largest animal taxon, with many species being agricultural and forest pests. The phylogeny of these species has aroused great interest among scientists. The characteristics of ovariole structure and ultrastructure are useful for phylogenetic work, especially with the improvement of micro technology in recent years. The types of ovarioles are varied. Usually, each family conforms to one type or another. However, in this study, we report on the ovaries of *Trypophloeus klimeschi* (Coleoptera: Curculionidae: Scolytinae), and find a different ovariole type from that of other known species of Curculionidae. We describe the features of the female reproductive system, ovary and oogenesis of *T. klimeschi* and also compare these features with those found in other Curculionidae. This study provides novel information on the reproductive biology of the Curculionidae.

**Abstract:**

The female reproductive system, ovary structure and ultrastructure of *Trypophloeus klimeschi* (Coleoptera: Curculionidae: Scolytinae) were investigated using light microscopy, scanning electron microscopy, and transmission electron microscopy. Its female reproductive system is comprised of two ovaries (each ovary has two ovarioles), lateral oviducts, common oviduct, spermathecal sac, spermathecal pump, two accessory glands and bursa copulatrix. Well-developed endoplasmic reticulum can be clearly seen in the secretory cells of spermathecal sac. This species has telotrophic meroistic ovarioles that are comprised of terminal filament, tropharium, vitellarium and pedicel. The terminal filaments are simple; each is comprised of cellular peritoneal sheath. The presence of several clusters of nurse cells in the tropharium is indicative that its ovarioles conform to the transition stage. This indicates that there are at least two different types (transition stage and secondary stage) of ovarioles in Curculionidae.

## 1. Introduction

Coleoptera is by far the largest animal taxon. It includes so many species associated with human production and living. The phylogeny of this order of insects has aroused great interest among scientists [1,2]. There are two main types of data used in phylogenetic work of Coleoptera: morphological data of exoskeleton and internal organs [3,4,5,6], and molecular data [7,8]. The female reproductive system is a traditional characteristic of internal organs [9,10,11,12,13,14,15,16,17,18]. Especially with the improvement of micro technology in recent years, the research of female reproductive system is moving into more microscopic areas, like the structure and development of ovarioles [19,20,21].

According to the presence or absence of nurse cells, the insect ovarioles can be divided into panoistic ovarioles and meroistic ovarioles. According to the positions of nurse cells, meroistic ovarioles can be divided into polytrophic meroistic or telotrophic meroistic. The telotrophic meroistic ovarioles are comprised of four parts: terminal filament, tropharium, vitellarium and pedicel. According to the structure of tropharium, these ovarioles can be divided into four subtypes: Ephemeroptera-type, Hemiptera-type, *Sialis*-type and polyphagan-type. The Ephemeroptera-type has linear clusters of nurse cells, one cell of which differentiates into an oocyte [22]. The Hemiptera-type consists of one cluster, in which posterior cystocytes develop as oocytes, all others as nurse cells [23,24,25]. The *Sialis*-type, that is known to occur in Sialidae (Megaloptera), Hydroscaphidae (Myxophaga) and Raphidioptera, emerges by cluster splitting during the multiplication phase of germ cells and of cluster fusion of all germ cells [23,26]. The polyphagan-type found in Polyphaga has a tropharium that is filled with moderately polyploid nurse cells and interstitial cells [23,27,28].

Based on the different extent of reduction of nurse cell membranes in polyphagan-type telotrophic meroistic ovarioles of diverse species, three basic types (in evolutionary order) of nurse cells organization could be established [20], representing tissues of a primary stage (characterized by intercellular bridges, combining all sister cells of one cluster, e.g., Coccinellidae, Elateridae, Staphylinidae, Tenebrionidae), transition stage (nurse cell membrane reduction is restricted to sister-cells, the content of free ribosomes between clusters of nurse cells may be same or different, e.g., Byturidae, Cerambycidae, Silphidae), and secondary stage (the cell membranes between nurse cells are totally reduced and there are, therefore, no differences in the content of free ribosomes, e.g., Bruchidae, Curculionidae, Scarabaeidae), respectively. It seems that each family conforms to one or other developmental pattern [20]. Curculionidae, with about 48,000 valid species, is the largest family of Coleoptera [29]. It may conform to the secondary stage, based on the research of only four species, as far as we know, namely, *Phyllobius urticae* (Entiminae) [20], *Anthonomus pomorum* (Curculioninae) [30], *Smicronyx fulvus* (Curculioninae) [31] and *Dendroctonus armandi* (Scolytinae) [15].

*Trypophloeus klimeschi* Eggers, 1915 (Curculionidae: Scolytinae) [32], is one of the most destructive pests of *Populus alba* var. *pyramidalis* (Bunge). It was first recorded in the Kyrgyz Republic, which borders Xinjiang Uygur Autonomous Region in China [32,33,34]. Following an outbreak in the Dunhuang in recent years [35,36,37,38], this beetle has caused huge economic and ecological losses.

In this study, we will report on the polyphagan-type telotrophic meroistic ovarioles of *T. klimeschi,* which conform to the transition stage. The features of the female reproductive system, ovary and oogenesis of *T. klimeschi* are also described and are compared with those of other species.

## 2. Materials and Methods

### 2.1. Insects

*T. klimeschi* (larvae and pupae) collected from the bark of infested *P. alba* var. *pyramidalis* in Dunhuang City (40°06′50.61″ N, 94°36′10.24″ E), Gansu Province, China, were reared in 24-hole plates with feed containing *P. alba* var. *pyramidalis* bark powder in an artificial climate incubator (14L: 10D, 25 ± 1 °C, 65 ± 5% relative humidity) [35]. According to our observations [35], female *T. klimeschi* generally began to lay eggs on the 12th day post-eclosion indoors and the 15th day post-eclosion in the wild. Eggs are about 200–300 μm in length and 150–200 μm in width. Indoors, adults of *T. klimeschi* can survive for up to 30 days after eclosion. Therefore, on the 1st, 8th, and 16th day after eclosion, 30 females were taken for later use in experiments. For anatomical analyses, the reproductive systems of 30 females—10 *T. klimeschi* each at 1, 8, and 16 days after eclosion—were observed with an OLYMPUS SZ2-ILST stereomicroscope and photographed with an OLYMPUS DP25 camera.

### 2.2. Scanning Electron Microscopy (SEM)

The reproductive systems of the 10 females in each age group were dissected and fixed in a solution containing 2.5% glutaraldehyde in 0.1 M phosphate (pH 7.2) for 12 h at 4 °C. The samples were washed in phosphate-buffered saline (PBS, pH 7.2), and post-fixation was performed in 1% osmic acid for 1.5 h at 4 °C. After three 10 min washes in the same buffer, the samples were dehydrated through a graded series of alcohol and isoamyl acetate; critical point dried with liquid CO_2_; and sputter coated with gold. Samples were then examined using a HITACHI S-4800 scanning electron microscope at 15 kV.

### 2.3. Transmission Electron Microscopy (TEM)

The 10 fixed samples of reproductive systems for each age group were rinsed with PBS, and post-fixation was performed in 1% osmic acid for 1.5 h at 4 °C. After five 10 min washes in the same buffer, the samples were dehydrated through a graded ethanol series and embedded in 14381-UC LR WHITE. Semithin sections were obtained with a glass knife on a LEICA RM2265 microtome, stained with toluidine blue, and observed with an OLYMPUS BX43F microscope. Ultrathin sections (70 nm thick) were obtained with a diamond knife on an ultramicrotome (LEICA ULTRACUT UCT), routinely stained with uranyl acetate and lead citrate, and observed with a HITACHI HT7700 transmission electron microscope.

## 3. Results

### 3.1. Gross Morphology of the Female Reproductive System

**Figure 1:**
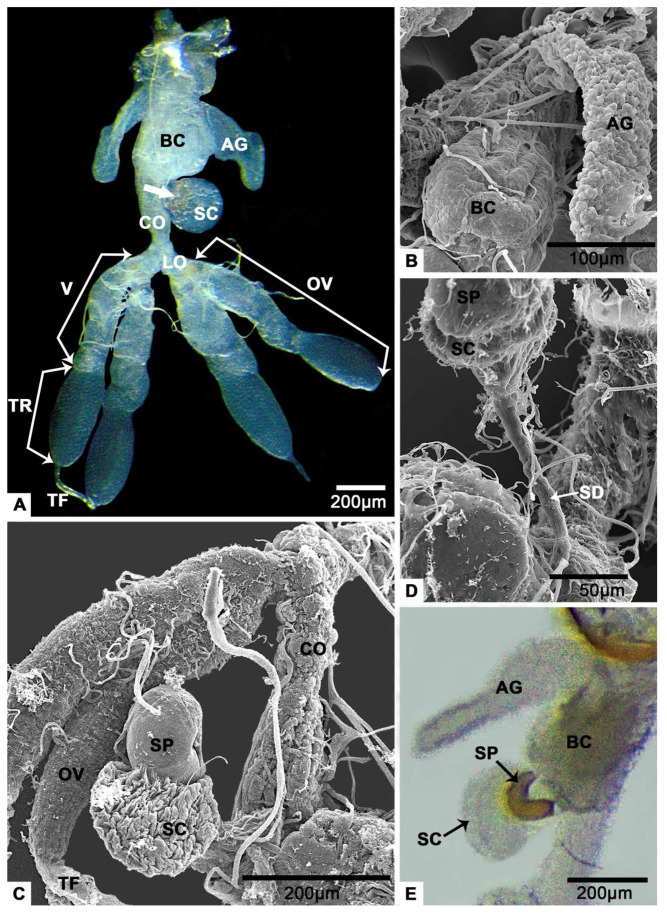
The female reproductive system of *T. klimeschi* on sixteenth day post-eclosion. (**A**) Light micrograph of the female reproductive system. The white arrow points to the spermathecal pump. (**B**) SEM micrograph of bursa copulatrix and accessory glands. (**C**) SEM micrograph of the female reproductive system. (**D**) The spermathecal duct. (**E**) Light micrograph of the crescent spermathecal pump and the spermathecal sac. Bursa copulatrix (BC); accessory gland (AG); spermathecal pump (SP); spermathecal sac (SC); spermathecal duct (SD); common oviduct (CO); lateral oviduct (LO); ovary (OV); vitellarium (V); tropharium (TR); and terminal filament (TF).

**Figure 2:**
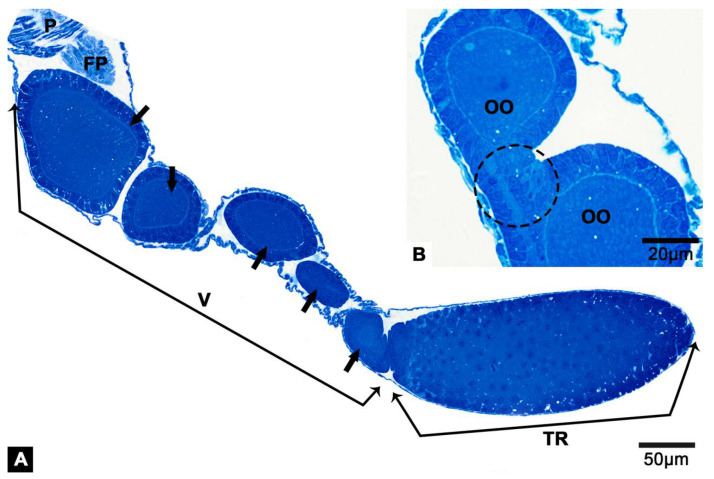
Logitudinal section of ovariole of *T. klimeschi* on sixteenth day post-eclosion. (**A**) Oocytes in successive stages of oogenesis (black arrows); (**B**) Adjacent oocytes are connected by follicular cells (dotted black circle). Vitellarium (V); tropharium (TR); oocyte (OO); follicular plug (FP); and pedicel (P).

**Figure 3:**
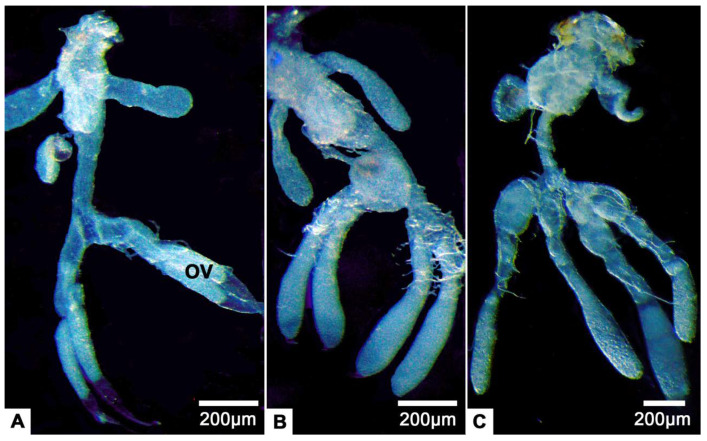
(**A**–**C**) The female reproductive system of *T. klimeschi* on the first, eighth and sixteenth day post-eclosion, respectively. Ovary (OV).

**Figure 4:**
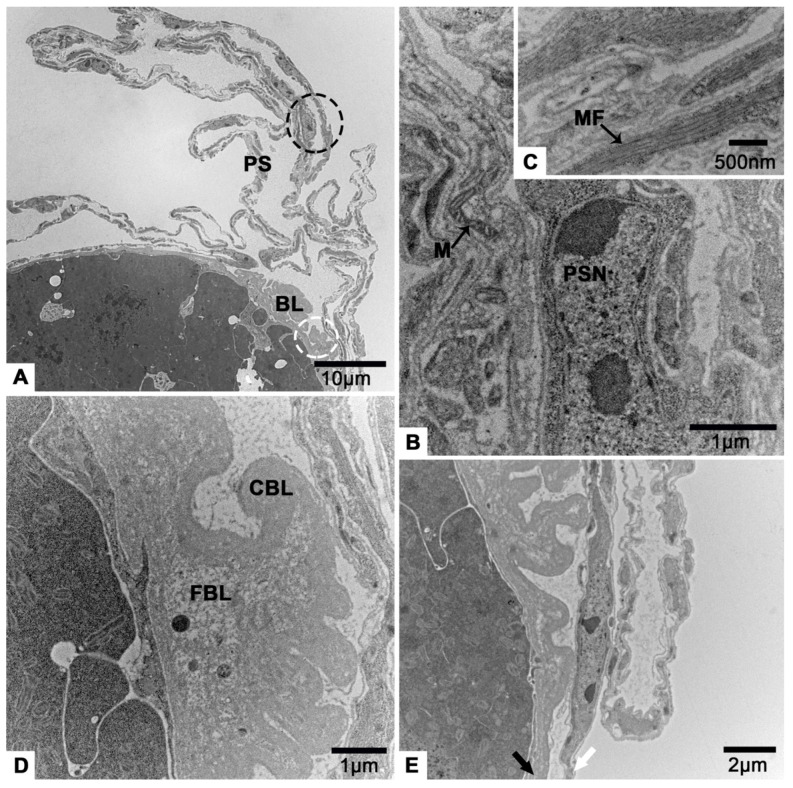
TEM micrographs of the terminal filament of the *T. klimeschi* ovariole on sixteenth day post-eclosion. (**A**) Longitudinal section of terminal filament. (**B**) An enlarged view of the area shown by the dotted black circle in Figure 4A. (**C**) The myofibrils of peritoneal sheath cell. (**D**) An enlarged view of the area shown by the dotted white circle in Figure 4A. (**E**) The peritoneal sheath (white arrow) and basal lamina (black arrow) become thinner as they extend to the posterior of the ovariole. Peritoneal sheath (PS); basal lamina (BL); nucleus of a peritoneal sheath cell (PSN); mitochondrium (M); myofibrils (MF); compact basal lamina (CBL); and fibrillar basal lamina (FBL).

**Figure 5:**
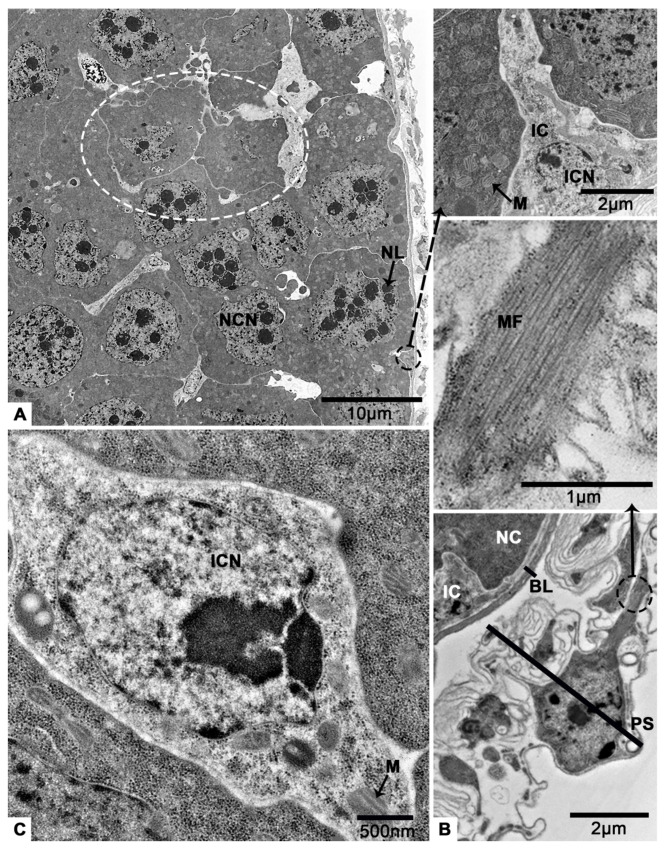

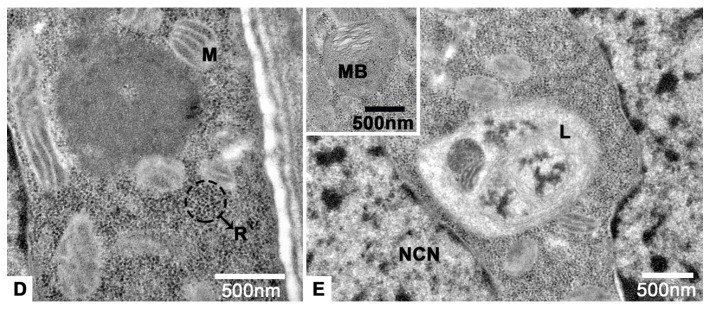
The anterior region of tropharium of *T. klimeschi* on sixteenth day post-eclosion. (**A**) Logitudinal section of the anterior region of tropharium shows several nurse cells clusters. In the dotted white circle, cell projections of nurse cell cluster extend in all directions are obvious. In the dotted black circle, an interstitial cell located next to the basal lamina is observed. (**B**) The peritoneal sheath and basal lamina. (**C**) The cytoplasmic contents of interstitial cell. (**D**,**E**) The cytoplasmic contents of nurse cell cluster. In the dotted black circle, the granular ribosomes are observed. Nurse cell (NC); nucleus of a nurse cell (NCN); interstitial cell (IC); peritoneal sheath (PS); basal lamina (BL); myofibrils (MF); lysosomes (L); nucleoli (NL); mitochondrium (M); nucleus of an interstitial cell (ICN); ribosomes (R); and multilamellar body (MB).

The female reproductive system of *T. klimeschi* is comprised of two ovaries, two lateral oviducts, a common oviduct, a spermathecal sac, a spermathecal pump, two accessory glands (Figure 1A) and a bursa copulatrix (Figure 1B). Each ovary has two telotrophic meroistic ovarioles. Each ovariole is comprised of terminal filament, tropharium, vitellarium and pedicel (Figure 1A and Figure 2A). From the first day to the sixteenth day post-eclosion, the ovarioles became thicker and longer, and the interior of the ovarioles also changed from slightly transparent to turbid (Figure 3). In the vitellarium, nearly spherical eggs of increasing sizes posteriorly are clearly visible (Figure 2A and Figure 3C). From outer to inner, each ovariole is embraced by the peritoneal sheath and acellular basal lamina (Figure 4E and Figure 5B).

### 3.2. The Terminal Filament

The terminal filament of *T. klimeschi* is comprised of extended and folded peritoneal sheath (Figure 4A). Peritoneal sheath is comprised of many elongated cells containing abundant myofibrils, elongated nucleus and mitochondria (Figure 4B,C). Basal lamina is an acellular structure and gathered at the posterior of terminal filament (Figure 4D) where the two different appearances of basal lamina are obvious (one is fibrillar, another is compact) (Figure 4D). Peritoneal sheath and basal lamina become thinner as they extend to the posterior of the ovariole (Figure 4E).

### 3.3. The Tropharium

In the region of tropharium and vitellarium, the peritoneal sheath cells have rounder nuclei, and basal lamina is thinner and more uniform (Figure 5B). Myofibrils are still obvious in the peritoneal sheath cells (Figure 5B).

**Figure 6:**
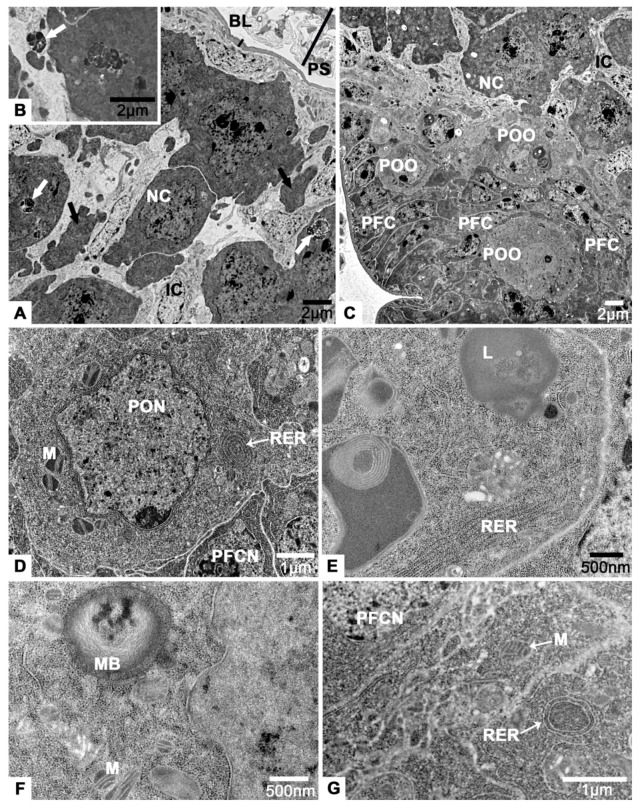
The posterior region of tropharium of *T. klimeschi* on sixteenth day post-eclosion. (**A**) The nurse cell clusters and interstitial cells. The remains of nurse cell cytoplasm are present in the interstitial cells (black arrows). There are many phagosomes (white arrows) filled with highly condensed material in the nurse cells. (**B**) Phagosomes in the interstitial cells (white arrow). (**C**) The preoocytes and prefollicular cells. (**D**–**F**) The preoocyte and its cytoplasmic contents. (**G**) The cytoplasmic contents of prefollicular cell. Nurse cell (NC); interstitial cell (IC); peritoneal sheath (PS); basal lamina (BL); preoocyte (POO); prefollicular cell (PFC); nucleus of a preoocyte (PON); nucleus of a prefollicular cell (PFCN); lysosomes (L); mitochondria (M); rough endoplasmic reticulum (RER); and multilamellar body (MB).

There are two distinct regions in the tropharium, the anterior region (Figure 5A) and the posterior region (Figure 6C). The anterior region is comprised of several nurse cell clusters and interstitial cells (Figure 5A). There are some individual nurse cells surrounded by their own cell membranes, but also other nurse cells that have fused into a syncytium, representing the transitional form of the tropharium (Figure 5A). Clusters of nurse cells are large and irregular, and their cell projections extend in all directions (Figure 5A). In the cytoplasm of nurse cell clusters, there are abundant free ribosomes, small round mitochondria, multilamellar bodys and lysosomes (Figure 5D,E). The nuclei of cluster are also irregular and contain several nucleoli (Figure 5A).

Interstitial cells are observed among the nurse cells. Both they and their nuclei exhibit varied shapes, although the latter are generally round (Figure 5A,C).

The posterior region of tropharium is comprised of nurse cell clusters, interstitial cells (Figure 6A), preoocytes and prefollicular cells (Figure 6C). However, compared to the anterior region, there are fewer nurse cell clusters and more and bigger interstitial cells, and the nuclei of nurse cells become more irregular and smaller (Figure 6A). There are many phagosomes filled with highly condensed material in the nurse cells (Figure 6A) and the interstitial cells (Figure 6B). The remains of nurse cell cytoplasm are present in the interstitial cells (Figure 6A). There are many mitochondria of different sizes, rough endoplasmic reticulum, lysosomes and multilamellar bodies in the cytoplasm of preoocytes (Figure 6D–F). In the cytoplasm of prefollicular cells, mitochondria and rough endoplasmic reticulum are observed (Figure 6G).

### 3.4. The Vitellarium

**Figure 7:**
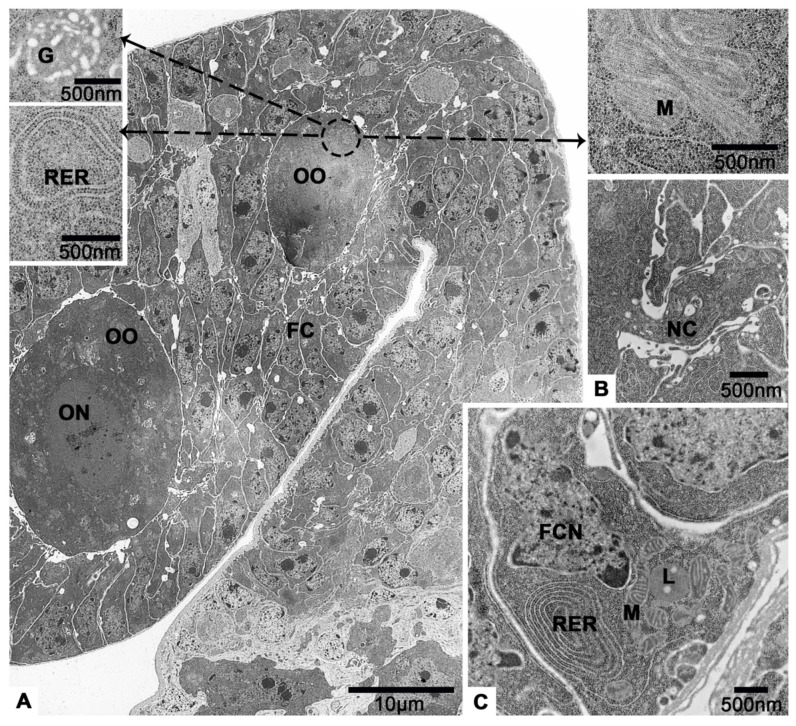
On sixteenth day post-eclosion, the oocytes and follicular cells of *T. klimeschi* in previtellogenesis. (**A**) The anterior region of vitellarium showing two developing oocytes. The magnified diagrams show the cytoplasmic contents of oocytes. (**B**) The nutritive cord. (**C**) A follicular cell and its cytoplasmic contents. Nucleus of an oocyte (ON); oocyte (OO); follicular cell (FC); Golgi apparatus (G); rough endoplasmic reticulum (RER); mitochondria (M); nutritive cord (NC); nucleus of a follicular cell (FCN); and lysosomes (L).

In vitellarium, oocytes are in successive stages of oogenesis and connected by follicular cells (Figure 2B and Figure 7A). In previtellogenesis, there are mitochondria, Golgi apparatus and endoplasmic reticulum in the cytoplasm of oocytes (Figure 7A). The cell membrane of the oocyte protrudes outward to form a nutrient cord (Figure 7B). Follicular cells accumulate irregularly around the oocyte (Figure 7A). Mitochondria, endoplasmic reticulum and lysosomes are observed in the cytoplasm of follicular cells (Figure 7C).

**Figure 8:**
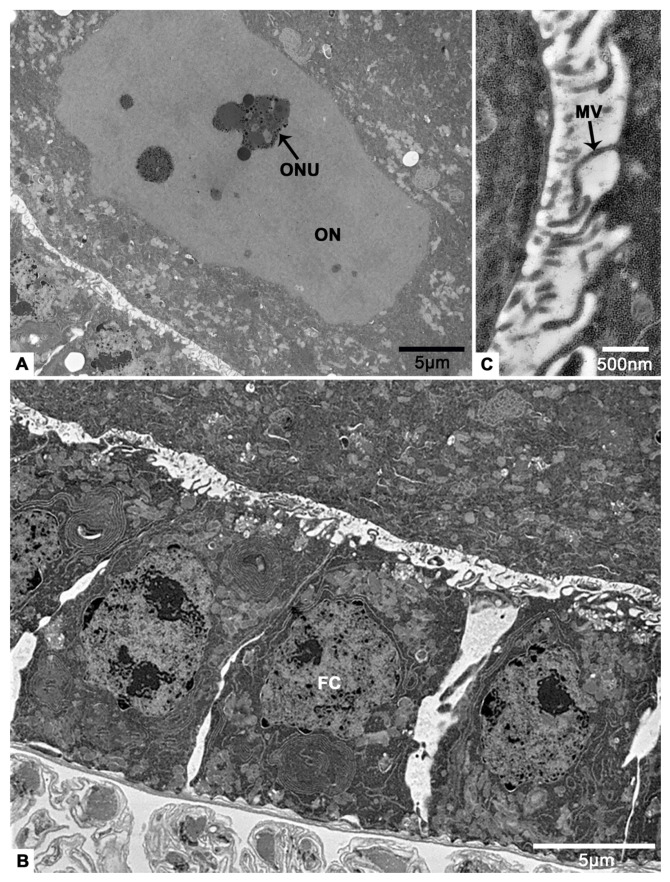

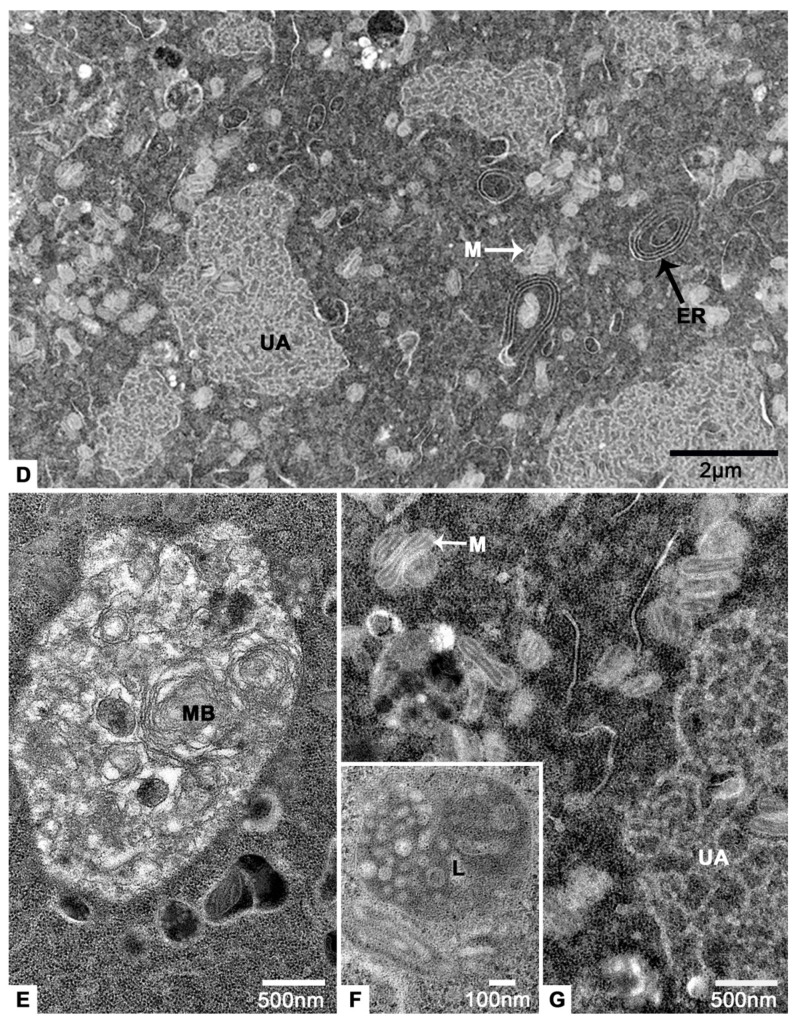
On sixteenth day post-eclosion, the oocyte and follicular cell of *T. klimeschi* in early vitellogenesis. (**A**) Oocytes become significantly larger. (**B**) Follicular cells become columnar and arranged in a monolayer. (**C**) The microvilli. (**D**–**G**) The cytoplasmic content of oocyte. Nucleus of an oocyte (ON); nucleoli of an oocyte (ONU); follicular cell (FC); microvilli (MV); mitochondria (M); endoplasmic reticulum (ER); multilamellar body (MB); lysosomes (L); and unidentified aggregates (UA).

In early vitellogenesis, the oocyte becomes larger and larger (Figure 8A). There is a layer of neatly arranged columnar follicular cells outside the oocyte (Figure 8B). Between them and the oocyte, abundant microvilli are observed (Figure 8C). The nucleoli of oocyte can be seen clearly (Figure 8A). In addition, there are many unidentified aggregates of low electron density, the endoplasmic reticulum, mitochondria, lysosomes and multilamellar body in the cytoplasm of oocytes (Figure 8D–G).

**Figure 9:**
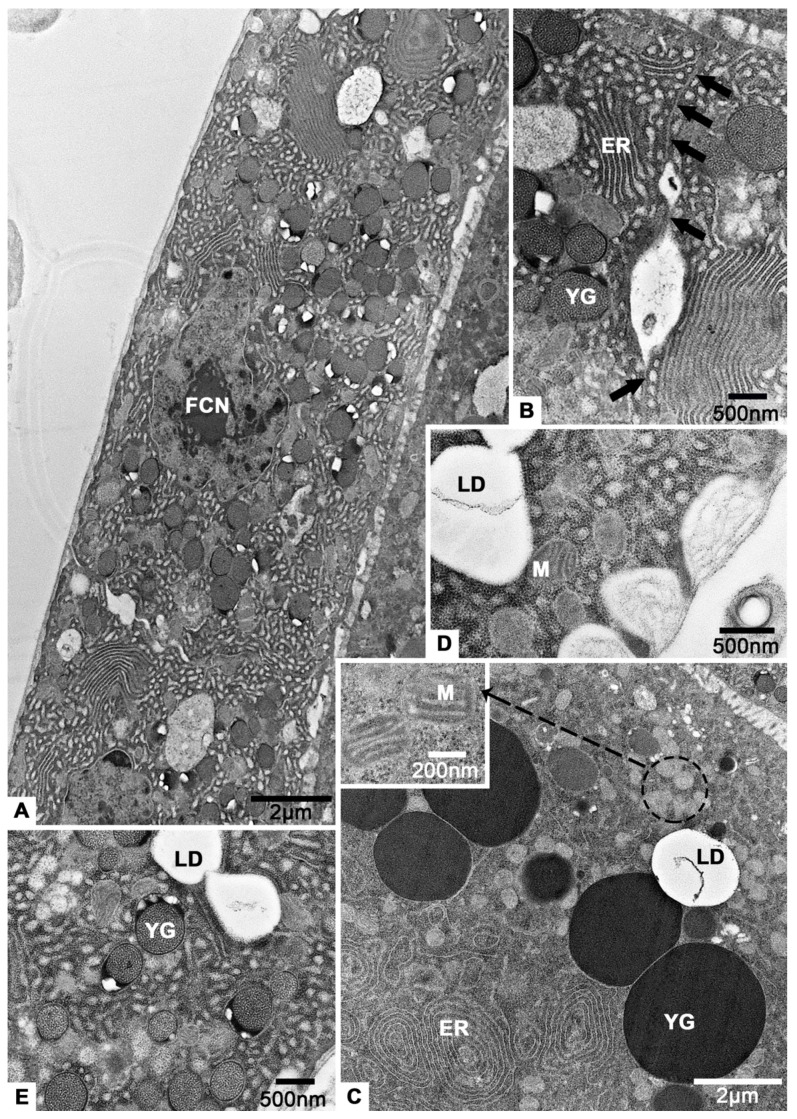
On sixteenth day post-eclosion, the oocyte and follicular cells of *T. klimeschi* in late vitellogenesis. (**A**) Follicular cells. (**B**) The gap between follicular cells (black arrows). (**C**) Cytoplasmic contents of an oocyte. (**D**,**E**) Cytoplasmic portions of follicular cells. Nucleus of a follicular cell (FCN); yolk granules (YG); endoplasmic reticulum (ER); lipid droplets (LD); mitochondria (M).

In late vitellogenesis, the follicular cells change from columnar to flat and the gaps between them almost disappear (Figure 9A,B). There are many yolk granules, lipid droplets, endoplasmic reticulum and mitochondria in the cytoplasm of oocytes (Figure 9C). The same phenomenon occurs in follicular cells (Figure 9D,E). However, the endoplasmic reticulum in follicular cells is very abundant and almost fills the entire cytoplasm (Figure 9A,B,D,E).

### 3.5. The Follicular Plug

**Figure 10:**
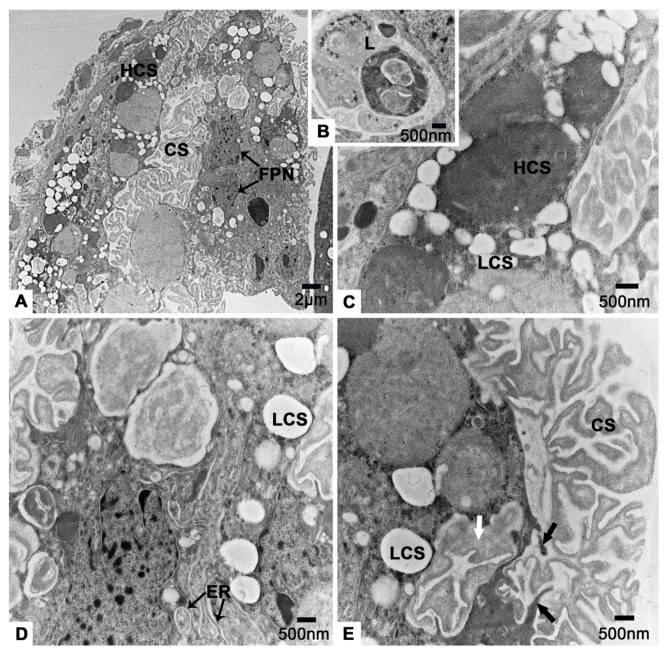
The follicular plug of *T. klimeschi* on sixteenth day post eclosion. (**A**) The follicular plug. (**B**–**D**) The contents of follicular plug. (**E**) The coralloid substances inside a follicular plug cell (white arrow) and being secreted from a follicular plug cell (black arrows). Nucleus of a follicular plug cell (FPN); endoplasmic reticulum (ER); lysosomes (L); low electron density circular inclusions (LCS); high electron density circular inclusions (HCS); and coralloid substances (CS).

The follicular plug is located posterior to the vitellarium of *T. klimeschi* (Figure 2A). This is a group of irregularly arranged cells with irregular nuclei (Figure 10A). There are many low electron density circular inclusions, high electron density circular inclusions, lysosomes and endoplasmic reticulum in the cytoplasm of these cells (Figure 10B–D). Around these cells, coralloid substances secreted by them can be observed clearly (Figure 10E).

### 3.6. The Spermathecal Sac and the Spermathecal Pump

**Figure 11:**
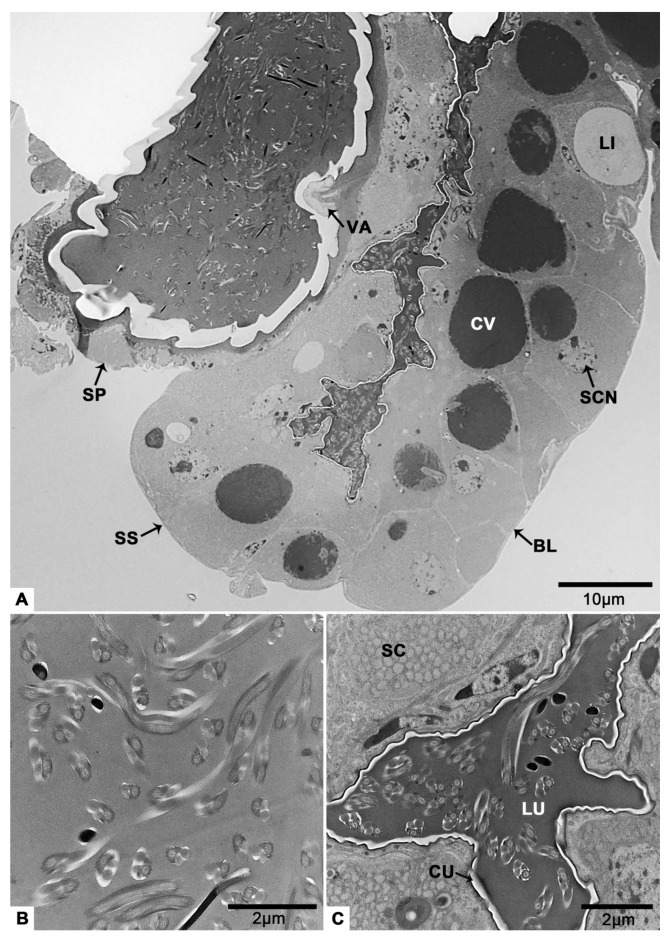

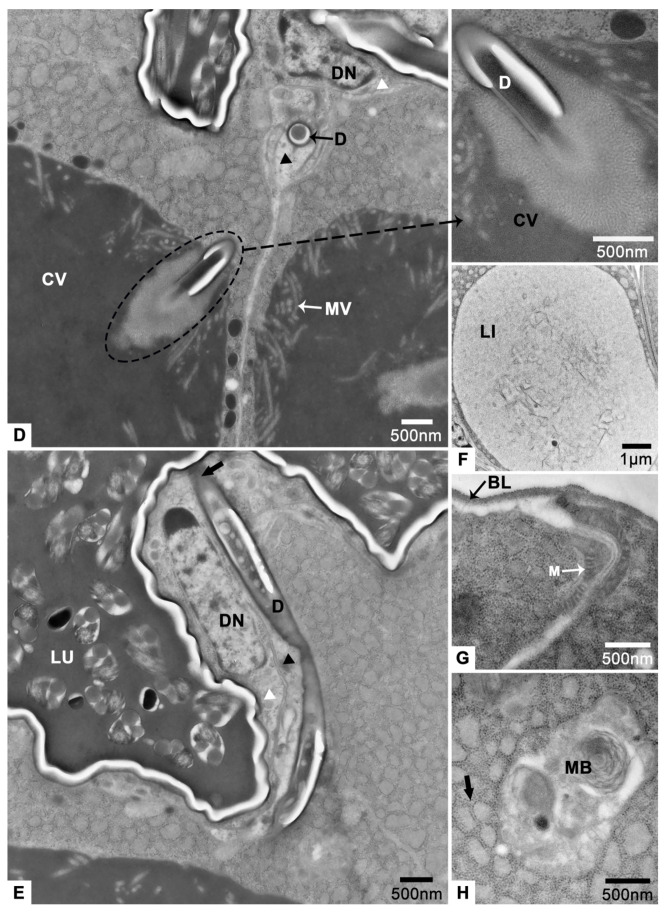
The spermathecal sac and the spermathecal pump of *T. klimeschi* on sixteenth day post-eclosion. (**A**) Longitudinal section of the spermathecal sac and the spermathecal pump, showing the valve that connects them. A large cavity bordered by microvilli is present in each secretory cell. (**B**) Sperm in the spermathecal pump. (**C**) Structure of the spermathecal sac, showing the presence of sperm in the lumen. (**D**,**E**) The cavity bordered by microvilli. In the dotted black circle, a duct protrudes from a secretory cell. The duct of secretory cell opening in the lumen (black arrow). The duct-forming cells (white triangles) are located next to the cuticle. The duct-forming cells (black triangles) are located next to the duct of secretory cell. (**F**) The light inclusion. (**G**) Mitochondria at the periphery of a secretory cell. (**H**) The reticulate endoplasmic reticulum (black arrow) and multilayer body. Spermathecal sac (SS); spermathecal pump (SP); valve (VA); nucleus of a secretory cell (SCN); basal lamina (BL); cuticle (CU); secretory cell (SC); light inclusion (LI); cavity (CV); nucleus of a duct-forming cell (DN); microvilli (MV); duct (D); lumen (LU); multilayer body (MB); and mitochondria (M).

The spermathecal pump is a light-yellow sclerotized sac (Figure 1E). It is next to the spermathecal sac (Figure 1C and Figure 11A) and connect with the junction of common oviduct and bursa copulatrix (Figure 1A) via a spermathecal duct (Figure 1D). The spermathecal sac and the spermathecal pump are linked by a valve (Figure 11A). There are sperm in both the spermathecal pump (Figure 11B) and the spermathecal sac (Figure 11C). Under a thin basal lamina (Figure 11A,G), the lumen of spermathecal sac is surrounded by an inner cuticle and an epithelium with secretory cells (Figure 11A,C). It is obvious that a duct protrudes from a secretory cell (Figure 11D). The duct opening in the lumen and secretions reach the lumen through the duct (Figure 11E). The duct is surrounded by the duct-forming cells that have thin cytoplasm reaching the apical region of the secretory epithelium (Figure 11D,E). Some duct-forming cells, in which very flat nuclei are observed, are located next to the cuticle for the responsibility for the production of the cuticle (Figure 11D,E). According to the longitudinal section, we know that there is at least one light inclusion in a secretory cell (Figure 11A,F). These masses are bigger than the nucleus (Figure 11A). The entire cytoplasm of secretory cell was almost filled with the reticulate endoplasmic reticulum (Figure 11H). Mitochondria are located at the edge of the cell (Figure 11G). There are also many multilayer bodies in the cytoplasm (Figure 11H).

### 3.7. The Accessory Glands

**Figure 12:**
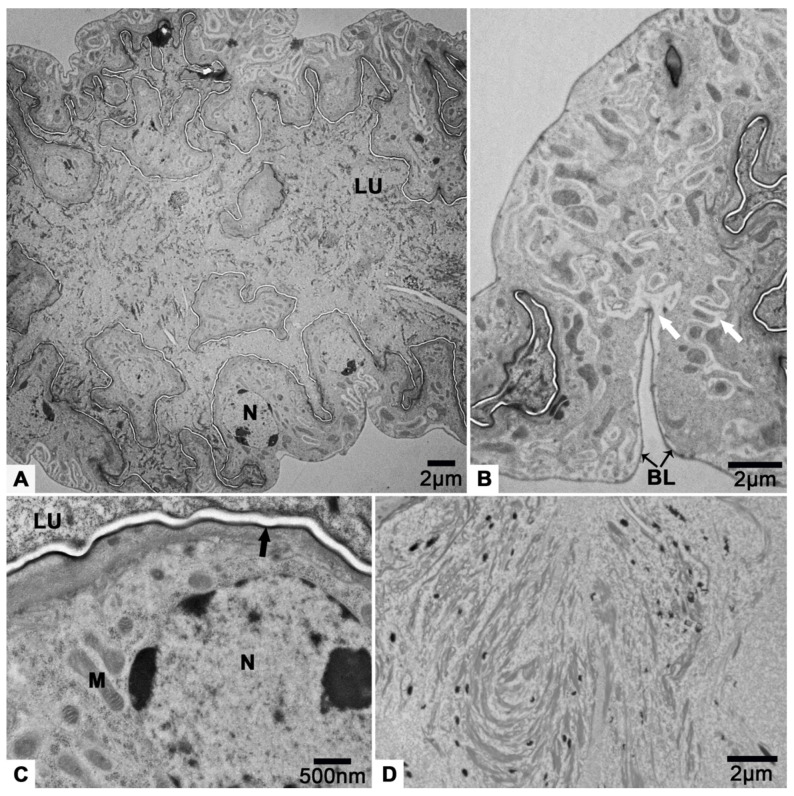
TEM micrograph of accessory gland of *T. klimeschi* on sixteenth day post-eclosion. (**A**) Cross section of the accessory gland. (**B**) The intercellular spaces (white arrows). (**C**) The inner surface of accessory gland (black arrow). (**D**) The flocculent substances in the lumen. Lumen (LU); mitochondria (M); nucleus (N); and basal lamina (BL).

The accessory glands are a pair of flat sacs located near the posterior end of the reproductive system (Figure 1A). Their outer and inner surface is uneven (Figure 1B and Figure 12A). Under a thin basal lamina (Figure 12B), many cells, that comprise the gland, with round nucleus (Figure 12A,C) are observed. Due to the extensive folding of the perimeters of the cells, the intercellular spaces of low electron density look irregular (Figure 12B). The inner surfaces produced by the cells are of high electron density (Figure 12C). There are mitochondria in the cells (Figure 12C). The lumen of the gland takes up a lot of space (Figure 12A), and flocculent substances are observed in here (Figure 12D).

## 4. Discussion

The most investigated female reproductive tract of Curculionidae [9,11,13] is comprised of two ovaries, lateral oviducts, common oviduct, spermatheca and bursa copulatrix. In addition, some Scolytinae, Erirhininae, Mecysolobini and Apioninae, and most Brentinae have accessory glands [9,13,14]. This study shows that the female reproductive tract of *T. klimeschi* is similar to that reported for other Scolytinae (Figure 1).

The accessory glands synthesis and secretion functions are always in concert with ovarian development and ovulation. The accessory glands produce a secretion that glues oviposited eggs to a substrate, hardens, forming a protective sheath encasing the eggs, or forms a protective plug sealing the buried eggs from the air [39,40,41]. The secretion consists, in whole or part, of secretory protein [42,43]. Correspondingly, the gland cell cytoplasm always contains extensive rough endoplasmic reticulum and Golgi apparatus [44,45]. The lack of extensive rough endoplasmic reticulum and Golgi apparatus in the gland cell cytoplasm of *T. klimeschi* (Figure 12) may suggest that secretion is not the main function of the accessory glands in this species. Its main function may be storage.

Spermathecae have been reported in all insect orders and there is one spermatheca in most studied insect species [46,47,48,49,50,51]. Its primary functional parts generally include the duct, reservoir and spermathecal gland. The glandular portion of the spermatheca generally consists of a few exocrine secretory cells [47]. This unit is either attached to the region between the duct and the reservoir [3,48,49] or consists of modified epithelial cells integrated into the reservoir wall [47]. The spermatheca of *T. klimeschi* is comprised of a spermathecal duct, a spermathecal pump and a spermathecal sac (Figure 1 and Figure 11A), as also observed in *Dendroctonus monticolae* [9] and *Dendroctonus ponderosae* [52]. Spermathecal sac in this study (Figure 11A,C) and spermathecal gland in other research have similar structure, being comprised of an epithelium with secretory cells surrounding a lumen bounded by an inner cuticle. They differ in that while there are sperm in spermathecal sacs (Figure 11A,C), there are apparently no sperm in spermathecal glands. Moreover, this study indicates that there is at least one large light inclusion in the cytoplasm of the secretory cells of spermathecal sac (Figure 11A,F). The light inclusion may be a storage form of secretions. However, in other species, secretions are more likely to be stored in smaller but more numerous vesicles [50,51].

It is known that all investigated Polyphaga have the polyphagan-type telotrophic meroistic ovarioles [19,20,21], as it occurs in the species studied here. According to the different extent of reduction of nurse cell membranes in ovarioles of diverse species, three basic types of nurse cells organization could be established [20], representing tissues of a primary stage, transition stage, or secondary stage, respectively. Additionally, it seems that each family conforms to one or other developmental pattern, with the Curculionidae conforming to the secondary stage [15,20,30,31]. However, in this study, the nurse cell membrane reduction is restricted to sister-cells, and there are no differences in the content of free ribosomes between different nurse cell clusters (Figure 5A). In other words, *T. klimeschi* conforms to the transition stage. Therefore, we think that the Curculionidae contains at least two developmental patterns of nurse cell organization. In addition, Büning’s research shows that the content of free ribosomes between clusters of nurse cells of the transition stage may be same or different [20].

The terminal filament of most Polyphaga is comprised of peritoneal sheath and somatic disc-shaped cells perpendicular to the long axis of the ovariole [3,16,53,54,55], while the Passalidae have a more complicated terminal filament having an enlarged proximal region with irregularly shaped cells embedded in a membranous system [16]. However, in *T. klimeschi* ovaries, the terminal filament was found to be simple, with only a cellular peritoneal sheath (Figure 4). Additionally, at the posterior end of the terminal filament, there is folded basal lamina that separates the terminal filament from the nurse cell organization (Figure 4). While in most Polyphaga, it is transverse septum that separates the terminal filament from the nurse cell organization [3,16,53,54,55]. Their transverse septum and basal lamina are always connected. We guess that the transverse septum is part of the basal lamina.

It is widely recognized that programmed cell death (PCD) is an evolutionarily conserved and genetically regulated form of cell death. It can be morphologically classified into three major types: apoptosis, autophagy and cytoplasmic cell death [56,57,58]. PCD plays a vital role in the insect oogenesis [28,59,60]. Its process has been studied in several orders, such as Diptera [61], Lepidoptera [62] and Hymenoptera [63]. In Coleoptera, all the three types of PCD are involved in the oogenesis of *Adalia bipunctata* (Coleoptera: Coccinellidae) [58]. Additionally, the remains of dead cells by autophagy have been found in the tropharium of the *Veturius sinuatus* (Coleoptera, Passalidae) [16]. In this study, in the posterior region of tropharium of *T. klimeschi*, there are more and larger interstitial cells, compared to the anterior region, in which the remains of nurse cells are present (Figure 6A). Additionally, many phagosomes filled with highly condensed material are found in the nurse cells and the interstitial cells (Figure 6A,B). All observations are evidence of the PCD in the tropharium of *T. klimeschi*. In addition, this may indicate that the increase of interstitial cells in the posterior region of tropharium of *T. klimeschi* is designed to assist nurse cells to complete the process of PCD.

## 5. Conclusions

The female reproductive system of *T. klimeschi* is comprised of two ovaries (each ovary has two ovarioles), lateral oviducts, common oviduct, spermathecal sac, spermathecal pump, two accessory glands and bursa copulatrix. This species has telotrophic meroistic ovarioles that are comprised of terminal filament, tropharium, vitellarium and pedicel. Its terminal filament is simple, only composed of cellular peritoneal sheath. The anterior region of the tropharium has several nurse cells clusters. This indicates that its ovarioles conform to the transition stage. In conclusion, the female reproductive system and the ovary structure of *T. klimeschi* are similar to that of other Curculionidae, with the exceptions that its terminal filament is comprised of cellular peritoneal sheath and it has ovarioles that conform to the transition stage.

## Data Availability

Not applicable.
